# Development of Sustainable Hydrophilic *Azadirachta indica* Loaded PVA Nanomembranes for Cosmetic Facemask Applications

**DOI:** 10.3390/membranes13020156

**Published:** 2023-01-26

**Authors:** Rizwan Tahir, Hasan B. Albargi, Adnan Ahmad, Muhammad Bilal Qadir, Zubair Khaliq, Ahsan Nazir, Tanzeela Khalid, Misbah Batool, Salman Noshear Arshad, Mohammed Jalalah, Saeed A. Alsareii, Farid A. Harraz

**Affiliations:** 1 Department of Textile Engineering, National Textile University, Faisalabad 37610, Pakistan; 2 Promising Centre for Sensors and Electronic Devices (PCSED), Advanced Materials and Nano-Research Centre, Najran University, Najran 11001, Saudi Arabia; 3 Department of Physics, Faculty of Science and Arts, Najran University, Najran 11001, Saudi Arabia; 4 Department of Materials, National Textile University, Faisalabad 37610, Pakistan; 5 Department of Dermatology, The University of Faisalabad, Faisalabad 38000, Pakistan; 6 Department of Chemistry, University of Sargodha, Sargodha 40100, Pakistan; 7 Department of Chemistry and Chemical Engineering, Lahore University of Management Sciences, Lahore 54792, Pakistan; 8 Electrical Engineering Department, College of Engineering, Najran University, Najran 11001, Saudi Arabia; 9 Department of Surgery, College of Medicine, Najran University, Najran 11001, Saudi Arabia; 10 Department of Chemistry, Faculty of Science and Arts at Sharurah, Najran University, Najran 11001, Saudi Arabia

**Keywords:** electrospinning, PVA nanofiber, *Azadirachta indica*, cosmetic, facial mask, biocompatible, membrane, antibacterial

## Abstract

Nanofiber-based facial masks have attracted the attention of modern cosmetic applications due to their controlled drug release, biocompatibility, and better efficiency. In this work, *Azadirachta indica* extract *(AI)* incorporated electrospun polyvinyl alcohol (PVA) nanofiber membrane was prepared to obtain a sustainable and hydrophilic facial mask. The electrospun *AI* incorporated PVA nanofiber membranes were characterized by scanning electron microscope, Ultraviolet-visible spectroscopy (UV-Vis) drug release, water absorption analysis, 2,2-diphenyl-1-picrylhydrazyl (DPPH) scavenging, and antibacterial activity (qualitative and quantitative) at different PVA and *AI* concentrations. The optimized nanofiber of 376 ± 75 nm diameter was obtained at 8 wt/wt% PVA concentration and 100% *AI* extract. The *AI* nanoparticles of size range 50~250 nm in the extract were examined through a zeta sizer. The water absorption rate of ~660% and 17.24° water contact angle shows good hydrophilic nature and water absorbency of the nanofiber membrane. The UV-Vis also analyzed fast drug release of >70% in 5 min. The prepared membrane also exhibits 99.9% antibacterial activity against *Staphylococcus aureus* and has 79% antioxidant activity. Moreover, the membrane also had good mechanical properties (tensile strength 1.67 N, elongation 48%) and breathability (air permeability 15.24 mm/s). *AI*-incorporated nanofiber membrane can effectively be used for facial mask application.

## 1. Introduction

Human skin comprises three-layered structures: the hypodermis, the dermis, and the epidermis [1]. The epidermal layer is the outermost layer exposed to the external environment and microbes for an extended period, imparting its aesthetics [2]. *Propionibacterium acnes* is the primary bacteria responsible for acne and pimples [3] on the face, which can be eliminated with natural antibacterial agents [4]. Initially, clay was used to overcome these flaws; however, poor penetration of the ingredient to the skin lowered its efficiency [5]. Other skincare products in use include creams, lotions, emulsifiers, and facemasks, the latter being the most likely. It functions as skin food, allowing the epidermal layer to heal more quickly and effectively in less time [6,7]. In the past, facemasks were made with more than 25 different chemicals [8], including mercury, bithionol, methylene chloride, and synthetic fragrances that were potentially harmful and infectious to people with sensitive skin [9]. Paola et al. used bacterial cellulose polymer to be used as a facemask. In vivo analysis of these facemasks showed improved skin lifting, smoothing, and anti-aging properties [10]. Nanotechnology has made it possible to increase the absorbency and efficacy of facemasks’ active ingredients as they have a large surface area and more entrapment sites [11,12,13]. Nanocapsules, nanocrystals, serums, and nano dendrimers are the most recent advancements in cosmetic facemasks, but they are expensive to produce and rarely available on the market [14]. Silver nanoparticles/guar gum-containing peel facemasks were synthesized and used for antimicrobial, anti-inflammatory, and antifungal activities. Results showed that this peel-off mask has significant antibacterial activity [15].

Among these nanomaterials, nanofibers have the most capability to integrate active substances in them at the nano level during their electrospinning process to get the inherited benefit [16,17,18,19]. Compared to conventional face masks, electrospun nanofiber membranes provide effective contact with the skin and release the active ingredients quickly and deeper into the skin pores. These membranes do not require preservatives to store the active agents and may be packaged as dry membranes, thus, minimizing the degradation rate of active agents due to non-aqueous storage [20,21]. Moreover, these membranes are developed from the green eco-friendly synthesis approach with natural ingredients as skin nutrients; therefore, they are the most suitable candidate for cosmetic applications [22,23]. On the other hand, nanofiber-based facemasks could eventually replace other methods due to their ease of manufacture and low cost via the electrospinning technique [24]. However, the end properties of a nanofiber can be altered by manipulating the parameters of the solution and machine [25]. Polyvinyl alcohol PVA/Chitosan/starch nanofibrous mats used as wound dressings exhibited superior cytocompatibility and antibacterial properties [26,27,28,29]. The development of a three-layered electro-spun polyvinyl alcohol/polycaprolactone/polyvinyl alcohol nanofibrous mat containing tetracycline hydrochloride (TC-HCL) and phenytoin sodium (PHT-Na) indicated that these mats exhibit exceptional swelling, antibacterial, and cell culture capabilities [30]. Mehta et al. modified the commercially available facemask composition to be electrospun to improve its moisturizing characteristics [31]. A dry facial mask containing *Huangshui* polysaccharide (cHSp), hyaluronic acid (HA), and polyvinyl alcohol (PVA) was fabricated by electrospinning with improved anti-oxidant activity and moisturizing effect [32].

Natural plant oils are very effective against various bacteria and could be used as a substitute for conventional antibiotics [33,34]. Since ancient times, different parts of organic plants have been used as antibacterial agents to fight against such bacteria [35]. Various solvent extracts of *Azadirachta indica (AI*) bark were examined for their antioxidant [36] and antibacterial activities [37], and the results showed that methanol and ethanol extracts had higher antioxidant capabilities than the other solvent extracts. Bi-layered nanofibrous mats (PVA and chitosan) loaded with *Azadirachta indica* were produced and checked for their antibacterial activity. Results indicated excellent antibacterial properties of developed mats, which can be potentially used as bio-medical material [38]. Research was conducted on various properties of nanofibrous mats having *Azadirachta indica* as an herbal antibacterial agent, which suggested the uniform diameter of nanofibrous mats and an antibacterial efficiency of 80% [39]. A simple, natural, and dry facial mask loaded with *Phyllanthus emblica* (*P. emblica*) was developed using an electrospinning technique. The proposed dry nanofiber facial masks are hydrophilic, biocompatible, and inflammation-free and exhibit superior tyrosinase suppression [40]. An electrospun nanofibrous membrane of PVA loaded with organic oils was produced for dermal applications. The composite nanofibrous membranes based on PVA comprise palmarosa oil and phytoncide oil, exhibiting outstanding antibacterial characteristics [41]. Bulus and his co-workers developed an electrospun cosmetic facemask consisting of aloe vera, black rice, and black cumin. The in vitro studies of the developed membrane showed excellent moisturizing and cell regeneration properties [42]. A composite nanofiber sheet of Polyvinyl Pyrrolidone/polycaprolactone (PVP/PCL) loaded with tea tree oil was developed with an electrospinning technique. The developed sheets possess effective antibacterial activity against *Staphylococcus aureus* and *Escherichia coli* (7.5 and 9.55 mm zone of inhibition), with up to 61% of antioxidant activity [43].

A few studies have been conducted with natural ingredients loaded on nanofibers for skin application. However, limited study has been explored on synthesizing *AI*-incorporated PVA nanofiber with effective and fast *AI* extract release. This research suggests an effective way to incorporate *AI* extract in PVA nanofiber during electrospinning, along with control release of *AI* extract when applying nanofiber membrane as a facial mask.

In this work, we prepared *AI* integrated PVA electrospun nanofiber membrane for a biocompatible facial mask. Nanofiber membranes based on different PVA and *AI* extract concentrations have been prepared through needless electrospinning. *AI* extract is integrated into nanofiber as a natural antibacterial agent, exhibiting effective antibacterial activity on the skin. Moreover, PVA is also a biopolymer and is recognized as a safe ingredient by the Food and Drug Authority, United States of America [44], providing a sustainable solution for various biomedical applications [45]. Fiber morphology and functional groups of nanofiber membrane were analyzed through the scanning electron microscope (SEM) and Fourier-transform infrared spectroscopy (FTIR). The *AI* extract release of the composite membrane has been analyzed through the UV-Vis spectrophotometer. The nanofiber membrane’s water absorption and contact angle have been estimated to evaluate the moisture management of the nanofiber membrane. The antibacterial activity and antioxidant characteristics are analyzed to calculate the functionality of the *AI*-incorporated PVA nanofiber membrane. Due to its effective drug release, biocompatibility, and porous structure, the as-prepared nanofiber can be used as a facial mask.

## 2. Materials and Methods

### 2.1. Materials

Polyvinyl alcohol (PVA) of Mw ~85,000–124,000 (99% hydrolyzed) and High-Performance Liquid Chromatography (HPLC) grade water were purchased from Sigma Aldrich, Taufkirchen, Germany. 2,2-diphenyl-1-picrylhydrazyl (DPPH) and ethanol were purchased from the local supplier of Alfa Aesar, Haverhill, MA, USA. Fresh leaves of *AI* were obtained from the biological gardens of The University of Agriculture in Faisalabad, Pakistan.

### 2.2. Extraction of AI Juice

The extraction of juice began with the collection of *AI* leaves. After thorough washing, the leaves were air-dried at room temperature for two hours. Then, the leaves were passed through a juicer machine and a strainer cloth to obtain juice which further passed through multiple stages of fine filtration processes. The filtered juice of *AI* was used purely as a solvent to dissolve the polymer in the case of samples with 100:0 *AI* concentration. However, the other samples, 75:25 (8P-75E) and 50:50 (8P-50E), were prepared through dilution of pure *AI* extract with HPLC water to get the required ratio.

### 2.3. Preparation of Electrospinning Solution

Electrospinning solutions were prepared by dissolving three PVA concentrations (6, 7, and 8 wt./wt.%) in a mixture of *AI* extract and HPLC water with different ratios, respectively. The concentration of *AI* extract was adjusted to 100:0 wt.%, 75:25 wt.%, and 50:50 wt.% of the solvent. These solutions were prepared with constant stirring at 600 rpm for 24 h at 60 °C.

### 2.4. Functional Nanofibrous Membrane Fabrication through Electrospinning

**Figure 1:**
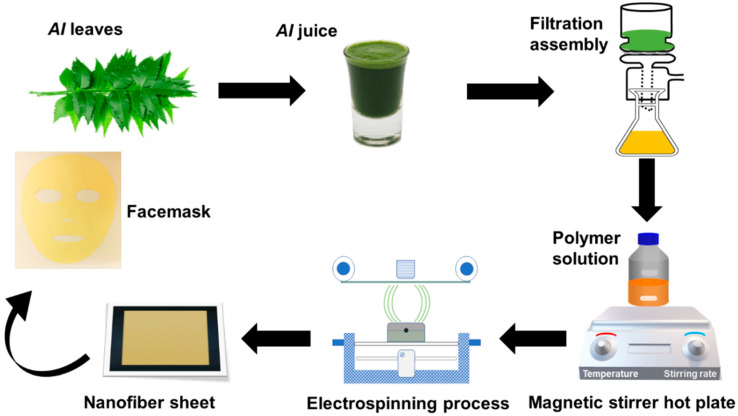
A schematic illustration of preparing *AI*-loaded PVA nanofibers facemask.

Figure 1 illustrates the process flow of the prepared nanofibers facemask, starting from the *AI* extraction from fresh leaves and solution preparation with PVA polymer. Subsequent electrospinning of PVA/*AI* extracts solution at needleless electrospinning setup (Elmarco Nanospider NSLAB, Liberec, Czech Republic, one spinning electrode, small carriage capacity 10 mL, spinning voltage 0–80 kV, and spinning distance 120–240 mm). After multiple trials, the process variables, such as applied voltage, spinning distance, and carriage speed, were held constant at 45 kV, 20 cm, and 25 mm/s, respectively. All solutions were run for 8 h to fabricate separate nanofiber sheets of 0.2 mm thickness. Environmental conditions (temperature 28 ± 2 °C and relative humidity 45 ± 3% R.H) were kept constant throughout the electrospinning process. The following combinations of membranes with three levels of PVA and *AI* extract were fabricated to analyze the impact of the PVA and *AI* extract concentration on the functional characteristics of the nanofibrous membrane, as given in Table 1.

### 2.5. Characterization and Techniques

SEM (MIRA 3 TESCAN, Kohoutovice, Czech Republic) was used to investigate the produced nanofibers’ fiber morphology. ImageJ software was used to analyze the diameter of prepared samples. The diameter of 100 fibers was recorded, and then the average diameter was calculated. Fourier transform infrared (FTIR) technique was used to investigate the functional group of the prepared *AI*-incorporated PVA-nanofibers membrane with an over a range of 400–4000 cm^−1^. It was performed on PERKIN ELMER Spectrum 2 (Waltham, MA, USA).

The particle size distribution of *AI* particles was determined by Zeta Sizer (Ver 7.11, Malvern, UK) using the dynamic light scattering (DLS) approach. The solution was sonicated in the water bath to prevent particle aggregation and disperse particles within the solution before the test. Single fiber tensile tester machine UTM-4 (Sonnenbergstrasse, Switzerland) measured tensile force and elongation at the break of prepared nanofibers according to standard ASTM D882-01. The sample size for testing was 5 mm × 50 mm. Each sample was tested five times, and the average was calculated. The air permeability of the developed nanofibers was measured on SDL ATLAS M-021A (Rock Hill, SC, USA) according to standard ISO-9237. The testing parameters were kept at 100 Pa pressure with a 20 cm head. Each sample was measured five times, and the average value was recorded. Each sample of nanofiber sheet was measured for its water contact angle (WCA) to confirm its hydrophilicity. The optical tensiometer (Theta lite/TL-100, Espoo, Finland) measured WCA via the sessile drop method. A sample with 1 × 1-inch dimensions was put on the sample tray, and the water was dropped onto the sheet’s surface. After monitoring the contact angle for 12 s, the machine recorded a final reading. The developed nanofiber membrane was cut into 2.5 cm × 2.5 cm pieces, and its dry weight, or *W_d_*, was noted at room temperature (30 °C and 55% R.h). After that nanofiber sheet was placed in PBS (0.01 M, pH 4.9–5.1) for different intervals of time (1, 3, 5, and 10 min), and the weight was recorded as *W_w_* after the extra water was wiped with a filter paper (blotted). The calculation for the water absorption rate was as follows in Equation (1).

(1) $$Water\;absorption\;\%age=\frac{W_{w}-W_{d}}{W_{d}}\times 100$$

Antioxidant tests for *AI*-loaded PVA nanofibers were conducted using a modified version of the DPPH radical scavenging assay described previously. An equal amount of PVA nanofibers integrated with *AI* immersed in a 3 mL ethanol-based DPPH solution of 10^−4^ M. Samples were kept at room temperature in the darkness for 60 min. Afterward, at 517 nm, the samples’ absorbance was measured using a UV-Vis spectrophotometer (Perkin Elmer, Waltham, MA, USA). The percentage of antioxidant activity was determined using the following Equation (2).

(2) $$\mathrm{Radical}\;\mathrm{Scavenging}\;\%\mathrm{age}=\left[\;\frac{\left(\mathrm{Abs}\;\mathrm{cnt}-\mathrm{Abs}\;\mathrm{smp}\right)}{\mathrm{Abs}\;\mathrm{cnt}}\;\right]\times 100$$

The sample of *AI* extract containing PVA nanofiber sheets was placed at 37 °C in 10 mL of potassium buffer solution (PBS, pH 4.9). At predefined intervals, 1 mL of each PBS was removed for additional analysis and substituted with 1 mL of PBS to maintain the release. UV-Vis spectrophotometer (Perkin Elmer, Model # λ 950) was set at a wavelength of 410 nm and used to study in vitro drug release. The calibration curve for *AI* extract was then used to convert the obtained absorbance into a concentration. Skin patch testing was performed at the Pakistan Council of Scientific and Industrial Research (PCSIR) site in Lahore. The skin patch testing was conducted in accordance with the Declaration of Helsinki, and approved by Ethics Review Committee at the Office of Research Innovation and Commercialization at National Textile University (AC/ORIC/20-43, 7 December 2021). Small patches of the created nanofibers were applied to sensitive areas (near the armpit) of the volunteer’s skin and monitored for irritation, sensitivity, and redness [46,47]. The sample size of 2.5 × 2.5 inches was placed in the armpit area of 30 volunteers (age group 25 to 40) and analyzed for various time intervals (10 min, 30 min, 1 h, 2 h, and 4 h) for skin patch testing as cited in the literature [48] and the number of volunteers varies according to the research study.

The antibacterial activity of the developed nanofibers was evaluated to check the efficacy against bacteria and the effect of PVA percentage and *AI* concentration on the bacteria by Agar disc diffusion test (qualitative) & Colony-forming unit (CFU) test (quantitative). In CFU, samples with varied *AI* concentrations (*AI*-50%, *AI*-75%, and *AI*-100%) having constant PVA percentage (8% wt/wt), and samples with varied PVA percentages (PVA-6%, PVA-7%-, and PVA-8%) with the same *AI* concentration (100% *AI*) were placed in a flask containing bacterial colonies. These flasks are then placed in a wrist shaker at 250 rpm overnight. Each flask underwent overnight shaking before being serially dissolved and placed in the incubator at 37 °C. The relative percentage of bacterial colonies was calculated from the flask with the test sample and the flask without the test sample. For the qualitative test, the antibacterial activity of samples was checked against the bacteria *S. aureus* samples (*AI*-50%, *AI*-75%, and *AI*-100%) and (PVA-6%, PVA-7%-, and PVA-8%) placed in Petri dishes with bacteria. Each sample’s zone of inhibition was assessed after the Petri dishes had been in the incubator for 24 h at 37 °C.

## 3. Results and Discussions

### 3.1. Surface Morphology

**Figure 2:**
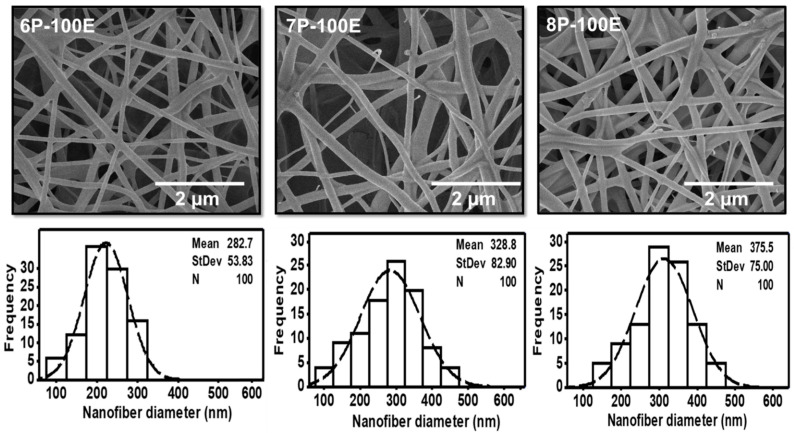
SEM images and histogram of diameter distribution of the developed electrospun nanofibers at 6, 7, and 8 wt/wt% of PVA, while using the 100% *AI* extract as solvent.

**Figure 3:**
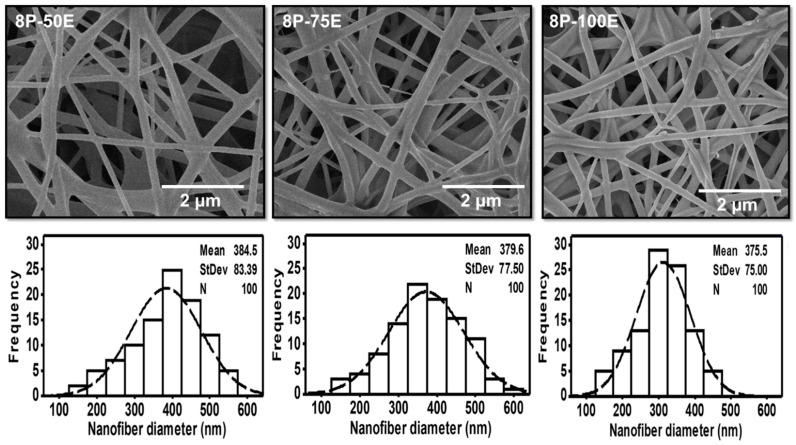
SEM images and histogram of diameter distribution of the developed electrospun nanofibers with different *AI*/water ratios 50:50, 75:20, and 100:0 while keeping the PVA concentration at 8 wt/wt%.

SEM analyzed all the optimized samples with different *AI* and PVA concentrations for surface morphology. Results showed that fibers are smooth, and no beaded structure is present in these samples, as shown in Figure 2 and Figure 3. The diameters of developed nanofibers are 283 ± 54, 329 ± 83, and 376 ± 75 nm for 6, 7, and 8 wt/wt% of PVA, respectively, while using the 100% *AI* extract, as presented in Figure 2. The PVA concentration has a direct relation and significant impact on the diameter of the nanofibers. The increases in the PVA concentration increased the nanofiber diameter, as a higher concentration of polymer enhances the entanglement of molecular chains, increasing the spinning solution’s viscosity. Hence the greater viscosity of the polymer solution leads to the formation of coarser nanofiber, having a greater nanofiber diameter. While at low polymer concentration, molecular entanglement is minimized, resulting in a less dense solution, and fibers with fine diameters are formed [49]. The histogram of nanofiber diameter at different PVA concentrations reveals that nanofibers with uniform diameter distribution were obtained at 8%, with a maximum load of the active agent by using 100% *AI* extract as solvent.

Figure 3 indicates the influence of the *AI* extract on the diameter of the PVA nanofibers at different *AI*/water ratios of 50:50, 75:25, and 100:0, whereas the PVA concentration is kept constant at 8 wt. The mean diameter is noted as 384 ± 83, 380 ± 76, and 376 ± 75, respectively, for the *AI*/water ratio 50:50, 75:25, and 100:0. The histogram of all the samples with different *AI*/water ratio reveals the uniform nanofiber diameter distribution. Hence, the impact of the *AI*/Water ratio on the nanofiber diameter is not as significant as PVA concentration, and no defined relation is noted between *AI*/Water ratio and nanofiber diameter.

### 3.2. Chemical Composition through FTIR & Particle Size and Distribution

**Figure 4:**
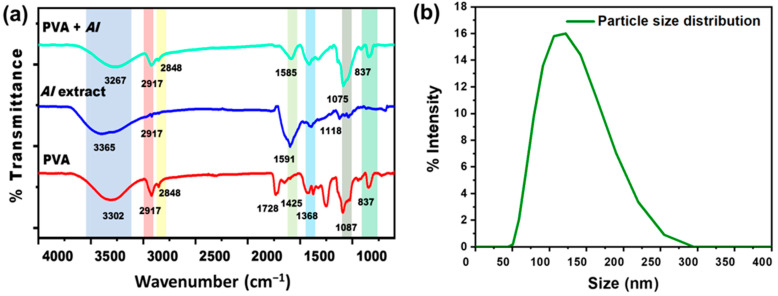
(**a**) FTIR spectra of PVA, *AI* extract, and *AI* incorporated with PVA nanofibers sheet (**b**) *AI* particle size distribution through DLS.

The FTIR Spectra of pristine PVA nanofibers, *AI* extract, and *AI*-incorporated PVA nanofibers are shown in Figure 4a. In PVA nanofiber, the broad transmittance peak at 3302 cm^−1^ is assigned to the hydroxyl group (O-H), the characteristic peak of pristine PVA nanofibers [26]. The peaks at 2917 cm^−1^ and 2848 cm^−1^ represent the asymmetric and symmetric CH_2_ stretching [50]. Due to the existence of unalcoholized acetyl groups, the peak around 1728 cm^−1^ was referred to be the result of carbonyl (C=O) stretching [51,52]. The presence of -CH_2_, -CH_3_, and C-O vibrational stretching is shown by the peaks at 1425 cm^−1^, 1368 cm^−1^, and 1087 cm^−1^, respectively [53]. In the IR spectrum of *AI* extract solution, the characteristic peaks at 3365 cm^−1^ and 2917 cm^−1^ are ascribed to stretching of O-H and vibrational bending of amine (N-H) groups due to polyols [54]. The peak at 1591 cm^−1^ is attributed to the C=C stretching of the alkene group, while the peak at 1118 cm^−1^ corresponds to the C-O stretching of triglyceride content of natural *AI* [55]. After blending *AI* extract with PVA, noticeably changed peaks have been observed in the PVA + *AI* nanofiber sheet spectrum. The peaks at 3267 cm^−1^ and 2917 cm^−1^ are ascribed to the O-H and N-H overlapping. The peak at 1585 cm^−1^ represents C=C stretching due to the alkane group in the structure of *AI* [56].

DLS result indicates the particle size distribution histogram in the range of ~50 nm to ~255 nm, as shown in Figure 4b, having the average particle/ingredients size of 123 nm [57]. This result indicates that *AI* nanoparticles can easily be incorporated into nanofiber sheets.

### 3.3. Mechanical Properties and Air Permeability Testing

**Figure 5:**
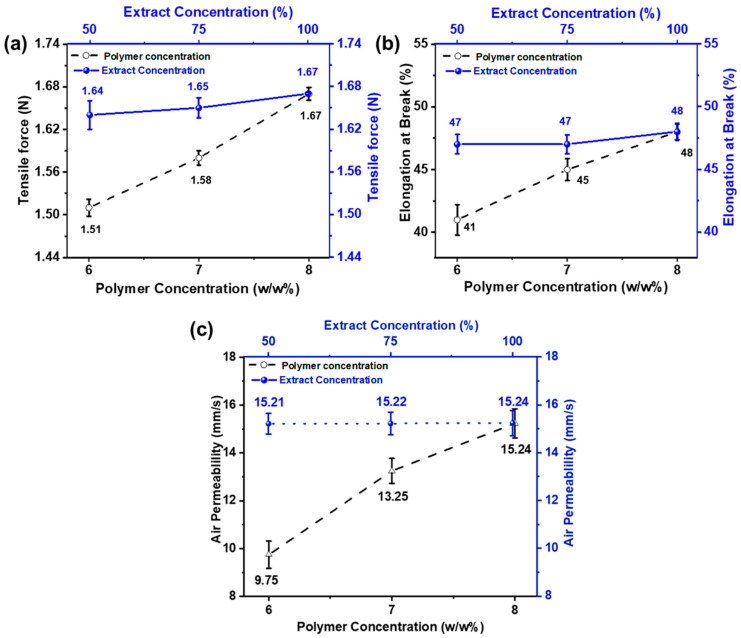
Effect of PVA wt./wt.% (6, 7, and 8 wt% with constant *AI*/water ratio 100:0) and *AI* concentrations (50, 75, and 100% with constant 8 wt% PVA (**a**) on tensile force (**b**) elongation at break (**c**) air permeability.

Tensile force and elongation at break were examined to analyze the mechanical properties of the nanofiber membrane. The effect of PVA concentration on mechanical strength has been noted, and the results are shown in Figure 5a. It can be noted that as the PVA concentration is decreased, mechanical strength is also reduced. This is because PVA tends to form nanofibers with finer diameters at lower concentrations. Additionally, during electrospinning, the larger percentage of solvent in the mixture tends to evaporate, leaving the polymer. Thus, the mechanical characteristics of electrospun PVA nanofibers decreased [58]. On the other hand, when the extract concentration is changed while keeping the polymer concentration the same, tensile force and elongation do not change noticeably. This showed that extract concentration did not affect elongation and tensile force.

Eichhorn and Sampson studied the relationship between fiber diameter and the pore size of nanofiber membranes. The role of fiber diameter in controlling pore size networks is significant [59]. The effect of electrospun nanofiber membranes on various properties, such as fiber’s size, and surface area diameter, was studied by Matsumoto et al. In biomedical and cosmetic applications, the open porous structure of nanofiber mats plays a vital role as it increases the effectiveness of nanofiber-based materials [60,61]. Because of a highly porous network and interconnected pores, nanofiber mats are considered ideal for such activities that provide an essential role in transporting oxygen and loaded nutrients to the skin. Figure 5c shows the air permeability of the developed electrospun nanofibers.

The result shows that the air permeability value increases as the fiber diameter increases. As the concentration of polymer increases, the gaps between the fibers also increase and vice versa, keeping the thickness of the nanofiber constant. In comparison, samples with different *AI* concentrations (50, 75, and 100%) show similar results because extract concentration does not affect the pore size and gaps between the nanofibers [62].

### 3.4. Hydrophobicity Study through Water Contact Angle & Swelling Behavior of the Developed Sheets

**Figure 6:**
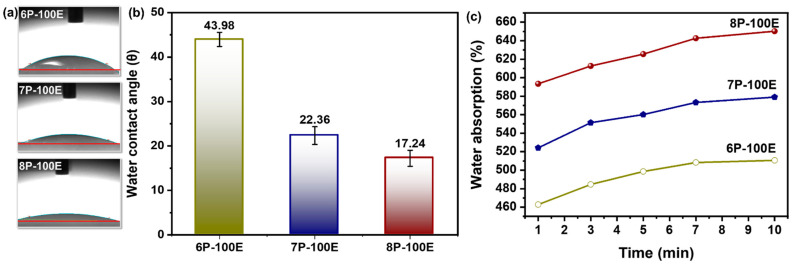
Hydrophobicity attributes of the developed nanofiber sheet (**a**,**b**) water contact angle through sessile drop method and its relation with PVA concentration with constant *AI*/water ratio 100:0 (**c**) water absorption rate of PVA nanofiber fabricated at 6, 7, and 8 wt% PVA with constant *AI*/water ratio 100:0.

The swelling percentage of nanofibers was much higher in all the samples studied because electrospun nanofibrous mats have a highly porous nature [63] and have higher surface energy [64]. The loaded drug molecules in the samples release it much more quickly and thoroughly to the desired environment due to the increased swelling. Because they are porous and hydrophilic [65], PVA nanofibers have the highest swelling percentages ranging from ~470 to ~660% as immersion time increases [66]. The PVA chains were tightly arranged before the test because they had been dried until their mass was consistent. The solution of PBS permeated the nanofiber sheet’s pore during the trial, causing into relaxing of the PVA chains [63]. Additionally, it is evident from Figure 6a,b that as PVA content rises; water absorption follows suit because PVA with higher weight percentages has more hydroxyl (-OH) groups, which increases water absorption [67].

The hydrophilicity and hydrophobicity of polymeric nanofibers play a significant role in practical applications [68]. Figure 6c illustrates the results of water droplet contact angle measurements on electrospun *AI*-PVA nanofiber surfaces. PVA’s hydrophilic nature demonstrates that as the PVA percentage increases, the (-OH) groups increase, resulting in a high affinity with water molecules, which gives nanofibers a higher moisture absorption capacity and a smaller contact angle [69]. As all samples have a contact angle of <50°, this indicates that the indigenous developed nanofibers are hydrophilic [70] and porous in structure [71].

### 3.5. In Vitro Drug Release Study & Radical Scavenging Activity through DPPH

**Figure 7:**
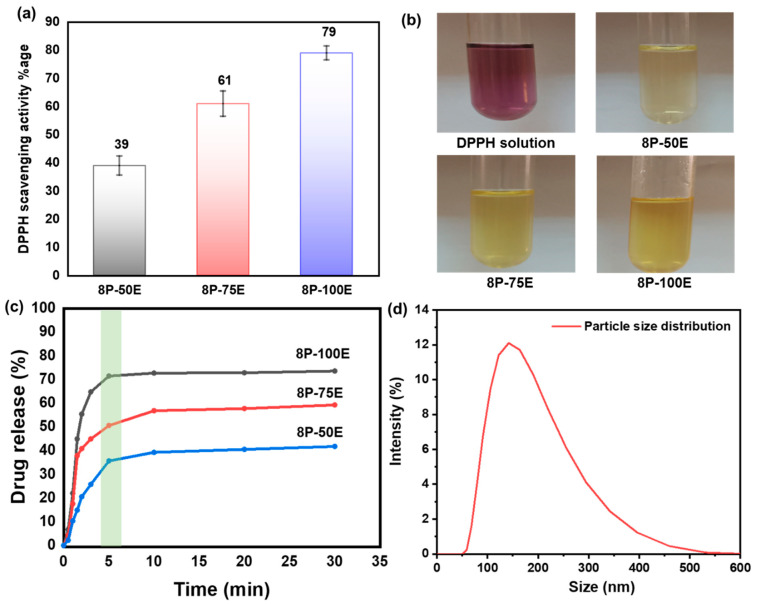
(**a**,**b**) DPPH free radical scavenging activity along with photographic results for the PVA nanofiber (**c**) drug release of the developed nanofibers sheet at different *AI*/water ratios 50:50, 75:25, and 100:0 with 8 wt%. PVA (**d**) *AI* particle size distribution released from the developed nanofibers membrane.

Figure 7a,b displays the DPPH test results for the free radical scavenging activity of an *AI*-loaded PVA nanofiber sheet.

Absorbance at 517 nm decreases when antioxidant molecules neutralize DPPH free radicals, turning them into a colorless byproduct. The results indicate that the anti-oxidant activity highly depends on the extract concentration in the samples; antioxidant activity increases as the extract concentration in the samples increases, and activity decreases as the extract concentration decreases [72].

The highest value of ~79% is noted for the sample 8P-100E, followed by ~61% and ~39% for the samples 8P-75E and 8P-50E, respectively. Electrospun nanofibers and liquid *AI* extract were studied for their in vitro release profiles during single medium dissolution. Since the pH of facial skin is between 4–6 [73], a PBS solution with a pH of 4.9 was chosen as the medium. Figure 7c shows cumulative drug release vs. time curves for samples 100, 75, and 50% at 1, 3, 5, 10, 20, and 30 min. 8P-100E showed burst release of more than 70% of the drug within 5 min. Similarly, 8P-75E and 8P-50E showed 50% and 35% *AI* nanoparticles release, respectively, within the 5 min of dissolution in PBS, followed by the linear pattern of drug release over the 30 min. The difference in the drug release percentage is due to the variation of extract loaded in the samples [74]. The burst release of drug is due to the high surface-to-volume ratio of nanofibers, as nanofibers tend to lower their surface energy immediately [75], the porosity of fibers [74], and the presence of drug particles near the fiber surface during electrospinning, which facilitates drug release [76].

The DLS technique was used to investigate the size of *AI* particles released from the nanofiber membrane of the developed sample, and it revealed *AI* nanoparticles having an average size of 144 nm, as indicated in Figure 7d.

### 3.6. Skin Patch Testing

The patch test is essential for identifying whether a particular cosmetic will cause an allergic or irritative reaction. The degree of response was measured by grading 0, +, ++, +++ for non-allergic, weak/low allergic, moderate allergic, and strong allergic, respectively [77]. The results written in Table 2 indicated no redness, irritation, or sensitivity, suggesting that the produced nanofiber sheet can be used safely on human skin [78].

### 3.7. In Vitro Antimicrobial Activities

**Figure 8:**
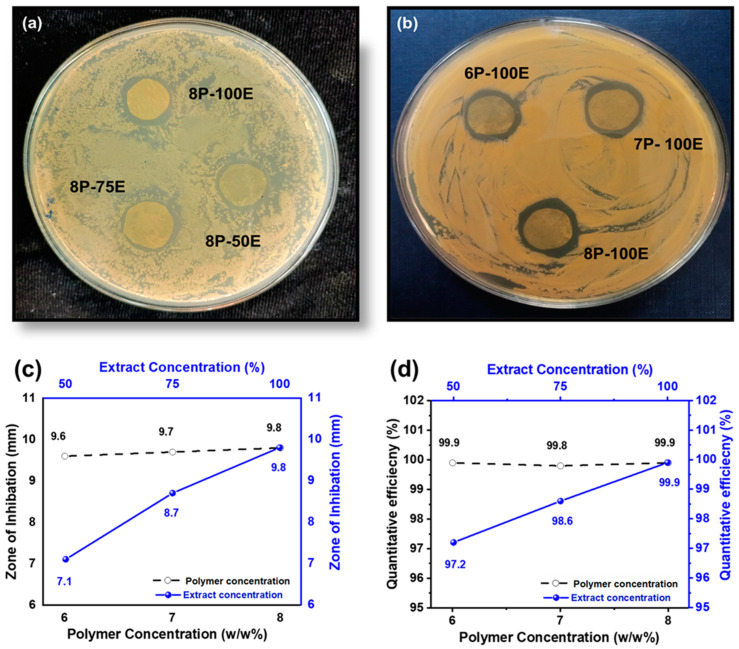
Effect of PVA wt./wt. %age and *AI* extract concentration (**a**–**c**) on the zone of inhibition through the disc diffusion method (**d**) quantitative efficiency via the colony-forming method.

**Figure 9:**
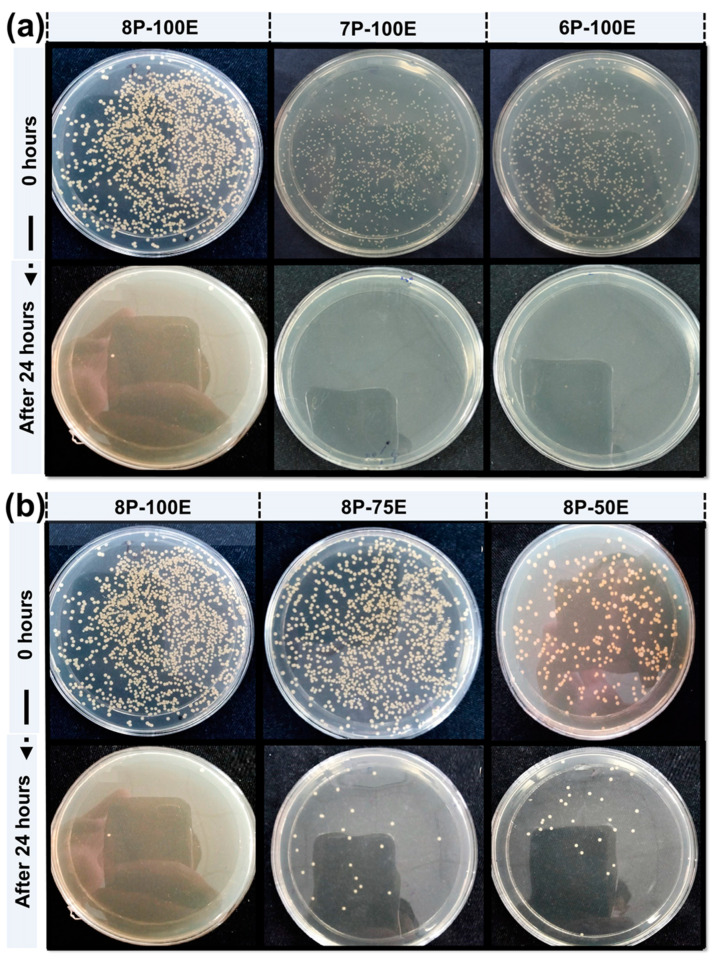
CFU results of the developed nanofiber sheets (**a**) samples with constant *AI* (100:0) concentration and different polymer concentration (**b**) samples with constant PVA percentage (8%) and varied *AI* concentration.

Bacterium is the primary cause of acne and pimples on the face, and *S. aureus* is one of the significant bacterias for acne [79]. Figure 8a,b and Figure 9a,b shows the visual representation of the samples’ qualitative and quantitative samples results against the *S. aureus* bacteria, respectively. Figure 8c shows the qualitative results that as the polymer concentration increases from 6% to 8%, the zone of inhibition changes unnoticeably from 9.6 mm to 9.8 mm, indicating that the change in polymer percentage does not affect the inhibition zone. In comparison, as the *AI* concentration increased from 50% to 100% in the samples, the inhibition zone expanded from 7.1 mm to 9.8 mm, demonstrating that increasing *AI* concentration enhances antibacterial properties [80].

In the quantitative antibacterial efficiency test, results are shown in Figure 8d. They indicated that as the concentration of *AI* increased in the samples from 50 to 100%, the efficiency percentage increased from 97.2 to 99.9%, showing that the *AI* extract concentration had an effect on the antibacterial efficiency. However, data showed that increasing the PVA percentage in the samples from 6 to 8 percent did not mitigate or improve the sample’s antibacterial effectiveness, indicating that the antibacterial effectiveness was independent of the PVA wt. (%) of the sample [81].

## 4. Conclusions

In this study, a biocompatible electrospun *AI*-integrated PVA nanofiber mask for facial skin remediation was developed. The composite nanofiber sheet comprises PVA nanofibers as carriers and *AI* nanoparticles as antibacterial skin agents. SEM images confirmed the fabrication of uniform nanofibers with a diameter from 282 to 375 nm at a 6–8% polymer percentage. The optimized nanofiber membrane, having a diameter of 376 ± 75 nm at 8 wt% PVA with 100:0 *AI*/water ratio, was used to evaluate the functional characteristics. According to the FTIR analysis, the successful incorporation of *AI* into PVA nanofibers was confirmed by the presence of their functional groups. Based on DLS analysis, *AI* ingredients loaded into nanofibers ranges from 50 to 250 nm. The nanofiber sheet also possesses good air permeability of 15.24 mm/s and tensile strength of 1.67 N, which improves with an increase in PVA concentration. The WCA of 43.98°, 22.36°, and 17.24° with the PVA concentration of 6, 7, and 8 wt%, respectively, indicate the hydrophilic nature of the membrane. The developed nanofiber sheets at 8% PVA of lowest WCA rapidly swelled via capillary force, reaching the highest swelling percentages of 660% after 10 min of soaking, whereas the nanofiber membrane with 6 and 7 wt% of PVA showed water absorption of 490 and 550%, respectively. The optimized nanofiber membrane also exhibits an excellent antioxidant activity of 79%, evaluated through scavenging of DPPH.

**Figure 10:**
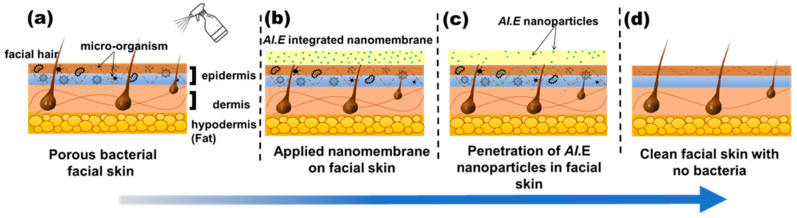
Working principle of the developed nanofiber facemask on facial skin. (**a**) wetting of facial skin with water (**b**) nanofiber membrane applied to the skin (**c**) penetration of *AI* nanoparticles in to skin pores (**d**) clean and bacteria free skin after removing the nanofiber membrane.

Furthermore, UV-VIS analysis shows that more than 70% of *AI* nanoparticles (drugs) are released in just five minutes for an optimized nanofiber membrane. The allergic patch test demonstrates that nanofibers have no adverse effects on the skin, such as redness, sensitivity, or irritation, proving their biocompatibility. The qualitative results showed the excellent antibacterial activity of the nanofiber sheet, whereas the quantitative antibacterial test confirmed its 99.9% effectiveness against *S. aureus*. Based on these functional characteristics, the best combination sample (8P-100E) with 8% of PVA and a 100:0 ratio of *AI*/water is recommended for further application/use. Hence, this innovative green *AI*-loaded nanofiber sheet can be applied as an effective facial mask, as demonstrated in Figure 10, delivering beneficial effects.

## Figures and Tables

**Table 1:** Nanofiber membrane samples at different PVA and *AI* concentrations.

Sr	Sample Code	PVA Concentration (wt%)	*AI* Extract Concentration (%)
1	6P-100E	6	100
2	7P-100E	7	100
3	8P-100E	8	100
4	8P-75E	8	75
5	8P-50E	8	50

**Table 2:** Results of skin patch tests.

Sample	Type of Allergy	Response
8P-100E (PVA 8% + *AI* 100%)	Redness	0
	Irritation	0
	Sensitivity	0

## Data Availability

Data will be provided on request.
